# Supplementary material for: Pharmacy-Based Opportunistic Atrial Fibrillation Screening at a Community Level: A Real-Life Study
Source: Healthcare (Basel). 2022 Jan 4;10(1):90. doi: 10.3390/healthcare10010090 (PMC8775917; doi:10.3390/healthcare10010090)
Supplement: Supplementary file 1 [file healthcare-10-00090-s001.zip › healthcare-1523793-supplementary.pdf]

Supplementary Table S1: Rate of screening according to age group population in the 3 communities

Age group	PESSAC			ARCACHON			SAINT MEDARD EN JALLES		
	Female	Male	All	Female	Male	All	Female	Male	All
65-69	17.8 (15.7-20)	11.8 (9.3-14.3)	15.2 (13.6-16.9)	26.7 (23.6-29.8)	22.8 (19-26.6)	25.1 (22.7-27.5)	14.7 (11.8-17.5)	13 (9.7-16.2)	13.9 (11.8-16)
70-74	24.6 (22-27.3)	17.7 (14.8-20.7)	21.5 (19.5-23.5)	38.8 (35.4-42.2)	32.4 (28.2-36.6)	36.1 (33.5-38.7)	31.9 (28.7-35)	17.8 (14.3-21.3)	24.8 (22.5-27.2)
75-79	18.4 (15.5-21.3)	16.3 (13-19.7)	17.6 (15.4-19.8)	25.8 (22-29.5)	24.5 (20.2-28.8)	25.2 (22.4-28)	19.8 (16.2-23.5)	17.7 (13.7-21.7)	18.9 (16.2-21.6)
80-84	12.8 (9.8-15.8)	14.3 (10.5-18.1)	13.5 (11.1-15.8)	12.5 (8.5-16.6)	15.7 (10.9-20.6)	13.8 (10.7-16.9)	14.5 (10.3-18.7)	13.3 (8-18.6)	14 (10.7-17.3)
85-89	10.3 (6.8-13.8)	13 (7.9-18.2)	11.1 (8.3-14)	8.1 (4-12.2)	10.2 (4.9-15.5)	8.9 (5.7-12.1)	13.9 (8.4-19.5)	12.7 (5.8-19.7)	13.5 (9.1-17.8)
90-	3.8 (0-8.6)	4.3 (0-11.4)	4.0 (0-7.9)	4.1 (0-8.7	8.3 (0-17)	5.0 (0.9-9.0)	3.8 (0-11)	13.4 (2.1-24.8)	6.3 (0.2-12.4)
>65	16.4 (15.2-17.6)	14.1 (12.6-15.5)	15.5 (14.6-16.4)	20.1 (18.6-21.6)	21 (19-22.9)	20.4 (19.2-21.6)	18.5 (16.9-20)	15.2 (13.4-17)	17 (15.8-18.2)

Rates of screening are expressed as percentage and 95% Confidence Intervals

Supplementary Table S2: Rate of unknown AF according to age group

	**65-69-year**	**70-74-year**	**75-79-year**	**80-84-year**	**85-89-year**	**≥90-year**
Negative screening, n	1200	1234	788	456	274	68
Positive screening of Unknown AF, n	3	17	13	11	8	1
Rate of Unknown AF, % (95%CI)	0,25 (0.06-0.79)	1,36 (0.82-2.22)	1,62 (0.90-2.34)	2,36 (1.24-4.34)	2,84 (1.31-5.79)	1,45 (0.07-8.98)

Rates of screening are expressed as percentage and 95% Confidence Intervals

AF: Atrial Fibrillation
